# MRI characteristics in treatment for cerebral melanoma metastasis using stereotactic radiosurgery and concomitant checkpoint inhibitors or targeted therapeutics

**DOI:** 10.1007/s11060-021-03744-4

**Published:** 2021-03-24

**Authors:** Maximilian Rauch, Daniel Tausch, Susanne Stera, Oliver Blanck, Robert Wolff, Markus Meissner, Hans Urban, Elke Hattingen

**Affiliations:** 1 Institute for Neuroradiology, Johann Wolfgang Goethe-University, Theodor Stern Kai 7, 60590 Frankfurt am Main, Germany; 2 Department of Radiation Oncology, Johann Wolfgang Goethe-University, Frankfurt am Main, Germany; 3 Saphir Radiosurgery Center, Frankfurt am Main, Germany; 4 Department of Dermatology, Johann Wolfgang Goethe-University, Frankfurt am Main, Germany; 5 Institute for Neurooncology, Johann Wolfgang Goethe-University, Frankfurt am Main, Germany

**Keywords:** Melanoma, Brain metastasis, Radiosurgery, Immunotherapy, Immune checkpoint inhibitors

## Abstract

**Introduction:**

Combination therapy for melanoma brain metastases (MM) using stereotactic radiosurgery (SRS) and immune checkpoint-inhibition (ICI) or targeted therapy (TT) is currently of high interest. In this collective, time evolution and incidence of imaging findings indicative of pseudoprogression is sparsely researched. We therefore investigated time-course of MRI characteristics in these patients.

**Methods:**

Data were obtained retrospectively from 27 patients (12 female, 15 male; mean 61 years, total of 169 MMs). Single lesion volumes, total MM burden and edema volumes were analyzed at baseline and follow-up MRIs in 2 months intervals after SRS up to 24 months. The occurrence of intralesional hemorrhages was recorded.

**Results:**

17 patients (80 MM) received ICI, 8 (62 MM) TT and 2 (27 MM) ICI + TT concomitantly to SRS. MM-localization was frontal (*n* = 89), temporal (*n* = 23), parietal (*n* = 20), occipital (*n* = 10), basal ganglia/thalamus/insula (*n* = 10) and cerebellar (*n* = 10). A volumetric progression of MM 2–4 months after SRS was observed in combined treatment with ICI (*p* = 0.028) and ICI + TT (*p* = 0.043), whereas MMs treated with TT showed an early volumetric regression (*p* = 0.004). Edema volumes moderately correlated with total MM volumes (*r* = 0.57; *p* < 0.0001). Volumetric behavior did not differ significantly over time regarding lesions’ initial sizes or localizations. No significant differences between groups were observed regarding rates of post-SRS intralesional hemorrhages.

**Conclusion:**

Reversible volumetric increases in terms of pseudoprogression are observed 2–4 months after SRS in patients with MM concomitantly treated with ICI and ICI + TT, rarely after TT. Edema volumes mirror total MM volumes. Medical treatment type does not significantly affect rates of intralesional hemorrhage.

## Introduction

Brain metastases are common in patients with melanoma. About 20% of patients with newly diagnosed melanoma have brain metastases, and in advanced metastatic melanoma, brain metastases are found in 44% of patients [1]. Overall median survival in these patients used to be just 4 months [2]. Hitherto existing therapeutic options were limited. Systemic chemotherapy is presumed to be ineffective due to low local drug concentrations owing to the blood–brain barrier [1]. Effective local treatments include surgery and radiosurgery for patients with a limited number of metastases [3, 4]. In contrast, the evidence for whole brain irradiation for disseminated disease remains inconclusive [5].

As treatment options for melanoma brain metastases remain challenging, they gained new impetus with the introduction of targeted therapies (TT) and immune checkpoint inhibitors (ICI). These include immunotherapy with interleukin-2, antibodies targeting programmed cell death protein 1 (PD-1) [Nivolumab, Pembrolizumab], antibodies targeting cytotoxic T-lymphocyte-associated protein 4 (CTLA-4) [Ipilimumab] and inhibition of the mitogen-activated protein (MAP) kinase pathway in melanomas with a V600-mutation using BRAF- [Vemurafenib, Dabrafenib, Encorafenib] or MEK-inhibitors [Cobimetinib, Trametinib and Binimetinib] [6].

Studies that investigated the combination of radiosurgery and TT or ICI in patients with melanoma brain metastases (MM) showed an improved survival over TT and ICI alone with median overall survival times ranging from 10.9 to 15.1 months [7–10].

To monitor treatment and detect associated complications in these patients, serial follow-up using magnetic resonance imaging (MRI) is performed. However, in patients undergoing radiosurgery and TT or ICI therapy it remains challenging as MRI findings are often difficult to interpret. Both immunomodulatory drug-induced mechanisms and radiation-induced changes may contribute to imaging findings that may simulate progressive disease [11, 12].

The aim of our study was to retrospectively investigate time course, time evolution and regression of MM as well as frequency of findings indicative of pseudoprogression in patients undergoing combined treatment with CyberKnife radiosurgery and ICI or TT.

## Material and methods

### Patient data

Data from patients with MM treated with SRS and immunotherapy were collected retrospectively from an institutional database. We included patients with MM that were treated with CyberKnife single-fraction robotic SRS between 2013 and 2017. Ethics approval was obtained by the ethics committee of the Johann Wolfgang Goethe-University Frankfurt. In total, 27 patients with 169 lesions were analyzed. Data collected included baseline demographics, UICC stage, BRAF (V-raf murine sarcoma viral oncogene homolog B1) and NRAS (neuroblastoma rat sarcoma oncogene) mutational status, number and localization of MM at the planning MRI scan, prior treatments, ICI type and cycles as well as TT type and cycles. Imaging was performed for treatment planning before SRS and at 2 months intervals thereafter. Follow-up period was 24 months.

### CyberKnife radiosurgery

Single session radiosurgery treatments were performed using the CyberKnife VSI Radiosurgery System (Accuray, Sunnyvale, USA). This system consists of a 6 MV linear accelerator mounted a computer-controlled robotic arm. Patient immobilization is achieved by a thermoplastic mask that is fixed to the treatment table. Single fractions ranged from 18 to 22 Gy (median 18 Gy). 21 patients received dexamethasone (4–8 mg) on the day of treatment to prevent from sudden brain edema.

### Immunotherapy

ICI were administered with at least three cycles with ipilimumab given intravenously at 3 mg/kg every 3 weeks, nivolumab at 3 mg/kg every 2 weeks and pembrolizumab at 2 mg/kg every 3 weeks. TT were administered on a daily basis with vemurafenib 240–960 mg/d, cobimetinib 20 mg/d, dabrafenib 300 mg/d and trametinib 2 mg/d.

### MR imaging

All scans were performed on a 1.5 T MRI system (Achieva 1.5 T, Philips Health Systems, Eindhoven, The Netherlands) using a 15-channel phased-array head coil. Images of the whole brain were acquired including the following sequences:

1. T1-weighted axial fast spin-echo (time repetition [TR] 664 ms, time echo [TE] 14 ms, slice thickness 5 mm, gap 0.5 mm, matrix 512 × 512, field of view [FOV] 230 mm^2^).
2. T2-weighted axial fast spin-echo (TR 8,000 ms, TE 120 ms, slice thickness 2.0 mm, spacing 2.2 mm, matrix 560 × 560, FOV 260 mm^2^).
3. T2*-weighted axial (TR 520 ms, TE 14 ms, slice thickness 5 mm, gap 5.5 mm, matrix 320 × 320, FOV 230 mm^2^).
4. 3D T1-weighted axial (TR 25 ms, TE 1.9 ms, flip angle 30°, section thickness 1.5 mm, matrix 256 × 256, FOV 210 mm^2^) after intravenous administration of single-dose gadolinium contrast agent (Gadovist, Bayer Vital, Leverkusen, Germany; 0.1 mmol/kg).

### Volumetric analyses

Lesions’ volumes were ascertained in isotropic T1-weighted contrast enhanced images using a semiautomatic edge detection tool (IntelliSpace Portal 11, Philips, Eindhoven, The Netherlands). Volumes of edemas were analyzed by volumetry of perilesional hyperintensities in T2-weighted images using the same software. Delimitable lesions with diameters < 1 mm were considered immeasurable in terms of measuring inaccuracy. The presence or absence of metastatic hemorrhages was ascertained by the presence of newly emerging hyperintensities on unenhanced T1-weighted and/or hypointensities on T2*-weighted images.

### Statistical analysis

Statistical analysis was performed using GraphPad Prism version 6 (GraphPad Software, San Diego, USA). Values are reported as the mean and standard deviation unless otherwise specified. Normal data distribution was ascertained using the D’Agostino-Pearson omnibus normality test. A paired Student’s *t* test was performed for the comparison of pre- and postoperative imaging series. An unpaired Student’s *t* test was used for all other comparisons. The Mann–Whitney rank-sum test was used for the comparison of non-normal distributed data, the Kruskal–Wallis-test for comparison of nominal scaled data. A *p* value < 0.05 was considered as statistically significant.

## Results

27 patients with a total of 169 MM (1–26 lesions per patient, mean 5.7) were identified. Patient data is summarized in Table 1.

**Table 1:** Patients’ demographics

Patients		
Sex	Female (no. of patients)	12
	Male (no. of patients)	15
Age at SRS treatment	Range (years)	30–85
	Mean (years)	61
UICC stage	2 (no. of patients)	2
	3 (no. of patients)	15
	4 (no. of patients)	10
MRI		
	No. of MM (range, mean)	1–26, 5.7
	Follow-up in months (range, mean)	2–48 (16.5)
Localization of primary tumor		No. of patients
	Scalp and face	3
	Trunk	5
	Upper extremity	2
	Lower extremity	6
	Vulva	1
	Unknown	4
Immunotherapy		(No. of patients/of MM)
ICI		17/80
	Ipilimumab	6
	Nivolumab	3
	Pembrolizumab	8
TT		8/62
	Vemurafenib	3
	Vemurafenib + Cobimetinib	1
	Dabrafenib	1
	Dabrafenib + Trametinib	3
ICI + TT		2/27
	Ipilimumab + Vemurafenib	1
	Pembrolizumab + Dabrafenib	1

Mutation status	Treatment	(No. of patients)
BRAF positive		12
	ICI	4
	ICI + TT	2
	TT	6
BRAF negative		15
	ICI	12
	ICI + TT	0
	TT	3
NRAS positive		4
	ICI	4
	ICI + TT	0
	TT	0
NRAS positive		23
	ICI	13
	ICI + TT	2
	TT	8

*SRS* stereotactic radiosurgery, *MM* melanoma metastases, *ICI* immune checkpoint inhibitor, *TT* targeted therapy

17 patients harboring 80 lesions received ICI, 8 patients with 62 lesions received TT and 2 patients with 27 lesions were treated with a combination of ICI and TT concomitant to SRS. In 3 patients that received ICI, 1 patient treated with TT, and 1 patient that received ICI + TT, medical therapy started 3–4 months preceding SRS, in all other patients, concurrent therapy with ICI and/or TT was administered within ± 4 weeks of SRS procedure.

Mean patient age at the time of SRS was 61 years. There were no significant differences regarding age between the groups. SRS planning target volume (PTV) doses per lesion did not differ significantly between patients that received ICI (median 20 Gy), TT (median 19 Gy) and ICI + TT (median 19 Gy). Patients had different antecedent treatments (> 6 months preceding SRS) including systemic chemotherapy (*n* = 3), interferone treatment (*n* = 9), dendritic cell-based vaccination (*n* = 1), and surgical resection of metastases outside the central nervous system (*n* = 15).

There were no significant differences between the groups regarding UICC stage (*p* = 0.49) and time from MM diagnosis to SRS (*p* = 0.60).

**Fig. 1:**
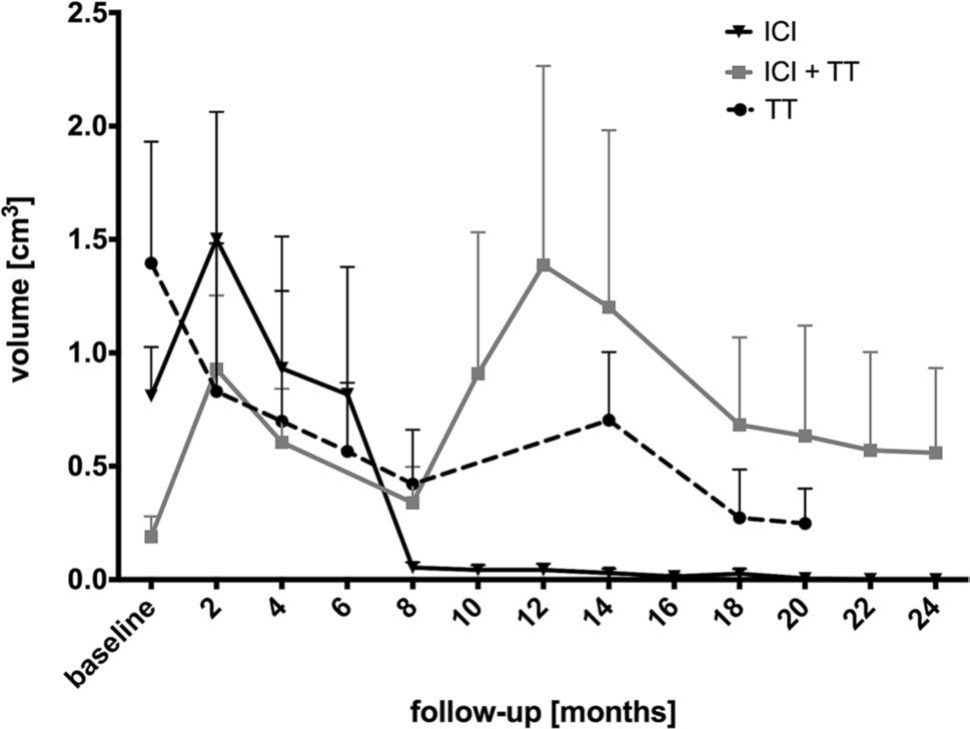
Time course of MM volumes depending on medical treatment. *ICI* immune checkpoint inhibitors, *TT* targeted therapy

After SRS, in patients treated with ICI, an early and significant (*p* = 0.028) increase in lesional volumes was detectable at first follow-up at 2 months (Fig. 1).

In this group, there were no significant differences between PD-1 and CTLA-4 ICI (*p* = 0.069). A later regression was seen at 4 months after SRS, with MM volumes not significantly different from baseline values.

In patients treated with ICI + TT, a significant volumetric increase of MM at 2 months (*p* = 0.043) and 4 months (*p* = 0.037) after SRS occurred, compared to baseline values, respectively. In patients treated with TT, MM showed an early volumetric regression (*p* = 0.004) (Fig. 1). Regarding individual MMs, in first MRI follow up 2 months after SRS, an increase in lesional volumes was seen in 25/80 MM (31.3%) treated with ICI, in 12/27 MM (44.4%) treated with ICI + TT, but only in 9/62 MM (14.5%) treated with TT.

There were no significant differences in volumetric response after SRS regarding lesion localization and initial MM volumes.

**Fig. 2:**
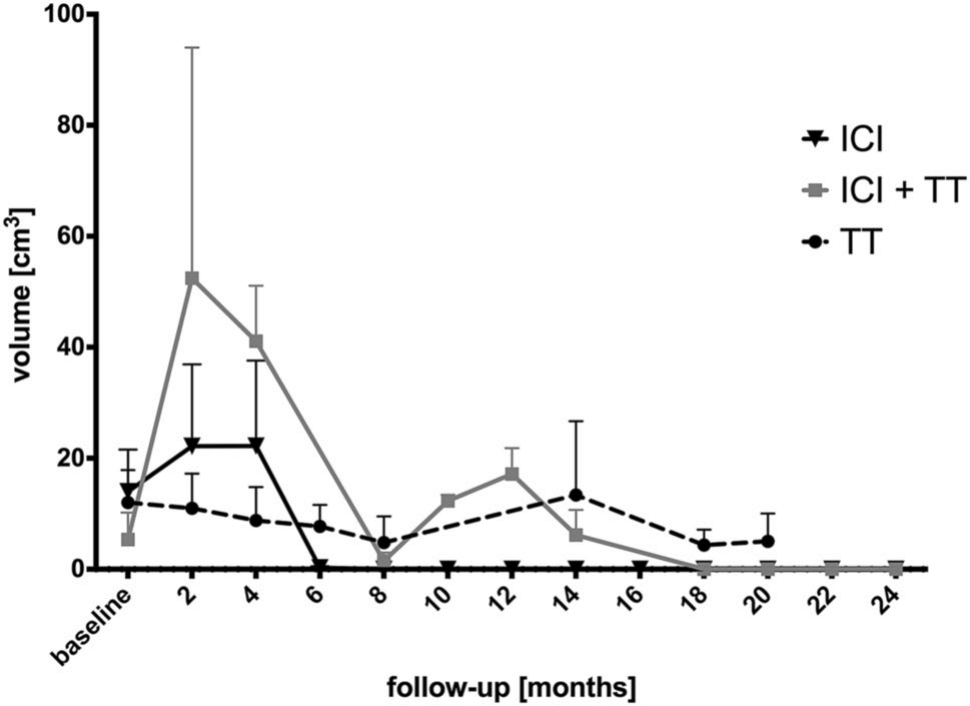
Total edema volumes in follow-up. *ICI* immune checkpoint inhibitors, *TT* targeted therapy

Total edema volumes moderately correlated (*r* = 0.57; *p* < 0.0001) with total MM volumes for any patient at given follow-up time points (Fig. 2).

In patients treated with ICI + TT, 23/27 (85.2%) metastases showed an increase in volumes and perifocal edema at 12 months follow-up after SRS compared with nadir at 8 months. Of these 10/23 (43.5%) showed an early reversible increase within the first 4 months.

In patients treated with TT, 50/62 (80.6%) metastases had an increase in volumes at 12 months follow-up after SRS compared with nadir at 8 months. In this group, none of these metastases showed early reversible volume increases.

**Fig. 3:**
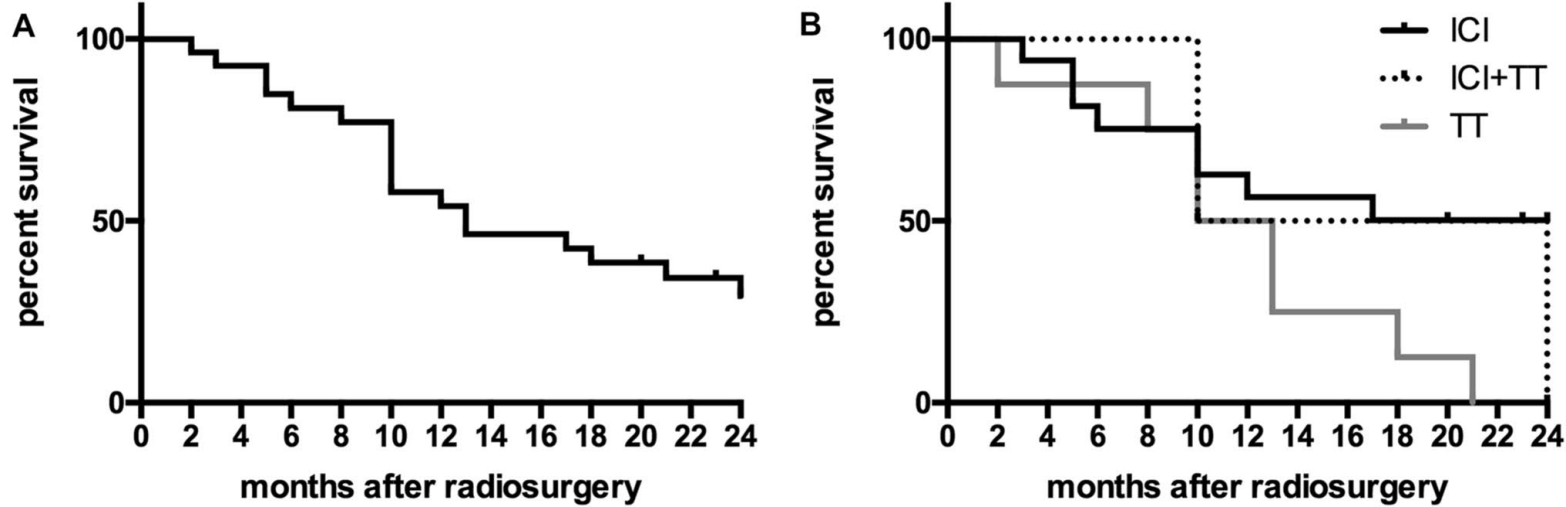
Kaplan–Meier-plots of overall survival (**a**) and survival regarding type of medical therapy after stereotactic radiosurgery (**b**). *ICI* immune checkpoint inhibitors, *TT* targeted therapy

Median overall survival from the date of SRS was 13 months. In patients receiving ICI + TT and TT, median survival was 17 and 11.5 months (*p* = n.s.), respectively. Median survival was not reached in patients treated with ICI within the 24 months follow-up period. Plots of overall survival and survival depending on medication are shown in Fig. 3.

**Fig. 4:**
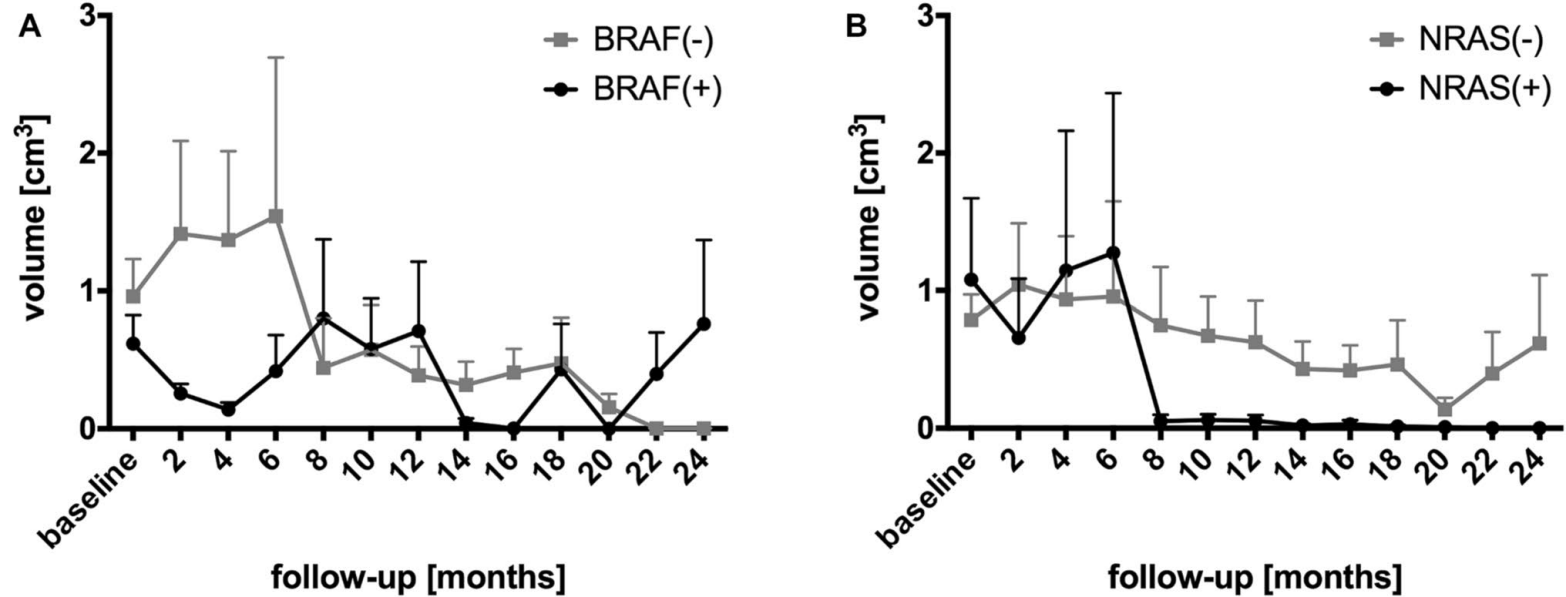
Time response in patients with BRAF (**a**) and NRAS (**b**) mutations

12 patients (44.4%) were positive for BRAF mutation and 4 (14.8%) positive for NRAS mutation. MMs with BRAF positive status showed an early volumetric regression after SRS (p = 0.038) compared to those with BRAF negative status (Fig. 4a). 4/12 (33.3%) BRAF positive patients were treated with ICI, 2/12 (16.7%) with ICI + TT and 6/12 (50%) with TT, respectively.

Up to 6 months after SRS, there were no significant differences in MM volumes between NRAS positive and NRAS negative patients (Fig. 4b).

10/169 MMs (= 5.9%) showed preexisting bleeds. Newly emerging metastatic bleeds in first follow-up after SRS occurred in 37/169 MMs (= 21.9%). There was no significant difference for any given group. In one case, bleeding led to a significant mass effect, necessitating surgical intervention. Bleeding with a longer interval to SRS occurred in 4 metastases at 3, 6, 8 and 24 months follow-up, respectively.

## Discussion

The aim of our study was to determine time evolution and incidence of MRI findings indicative of pseudoprogression in the monitoring of MM treated with CyberKnife SRS and concomitant ICI or TT.

In general, SRS is considered as the primary form of radiation therapy for patients with a limited number of small to medium-sized brain metastases [3, 4]. However, small metastases that are invisible on treatment planning imaging are accordingly not irradiated. Combinations of SRS and TT or ICI have been used to overcome this limitation as they may also affect neoplastic cells outside irradiated volumes.

Sole SRS was shown to affect distant metastases also without concomitant immunotherapy. Even when its mechanisms are not fully understood, it is based on the release of cytokines and tumor cell antigens within irradiated metastases that stimulate a cytotoxic immune response [13]. It not only affects remaining tumor cells locally, but also acts on distant metastases [14]. This phenomenon is known as the ‘abscopal effect’ [15]. Investigations in mice and humans suggested that in CNS neoplasms, the abscopal effect be potentiated by ICI based on amplification of cellular immune mechanisms [16, 17].

Studies in patients with MM comparing radiosurgery and ICI or TT to radiosurgery alone showed that combined therapies have the potential to increase response and local control rates compared to sole radiosurgery [18–22]. However, interpretation of imaging findings in patients undergoing combined therapy may be challenging as treatment with both SRS and ICI or TT is known to have the potential to generate findings that may confuse with progressive disease, so called pseudoprogression. Pseudoprogression generally is indistinguishable from true progression in a single MRI study. It is diagnosed by serial imaging when findings remain stable or resolve without changing the therapy regime.

It is known that MM may possibly show a delayed response after immunotherapy, especially after administration of ipilimumab, thus immune-related Response Criteria (irRC) were introduced [23, 24]. These require the initial increase of at least 25% in lesion load to be confirmed by follow-up imaging at least 4 weeks later to diagnose progressive disease and exclude pseudoprogression. We therefore analyzed time dependent changes and MRI characteristics of treated lesions over a long time period up to 24 months.

In our study, in patients treated with ICI and ICI + TT, an early progression of MM volumes indicative of pseudoprogression was seen 2–4 months after SRS.

Even though in several patients medical therapy was initiated more than 6 weeks preceding SRS, the finding is plausible as delayed effects of immunotherapy with pseudoprogression up to 6 months after initiation have been described [25]. In contrast, pseudoprogression was also reported in patients in whom drug therapy was initiated within 6 weeks after SRS [26].

In our investigation, the finding of increased MM volumes after SRS however, was absent in patients that received TT simultaneously to SRS. This group showed a significant reduction in overall MM volumes starting at 2 months follow-up.

Patel et al. [27] investigated size changes in brain metastases of different primary tumors after sole radiosurgery and observed an increase in metastasis volumes in about one third of cases during the follow-up period beginning at 6 weeks post radiation ranging up to 15 months. Lesions’ volumes showed transient increases of 3.6% after 12 months and 11.6% after 15 months, respectively. In contrast, a recent study reported that 20% of MM post SRS had a temporary, reversible increase in size much earlier at 3–6 months after concomitant treatment with anti-PD-1 ICI, compared to 5% with radiosurgery alone [28].

Radiation necrosis is another important side effect of radiation therapy that is associated with an increase in lesional volume. Compared to pseudoprogression, radiation necrosis usually occurs later with a maximum of these changes reported to be reached after 12–18 months [27].

We observed a second increase in lesional volumes and perifocal edema volumes that peaked at 12–18 months after SRS in patients that received TT and ICI and TT. However, there was no clear association of this phenomenon with the occurrence or absence of early volume increase after SRS.

Corticosteroids have long been the mainstay for the treatment of radiation necrosis in brain metastases. They decrease inflammatory signals and cytokines produced by the necrotic tissue thereby reducing the leakiness of the blood–brain barrier, leading to a swift decrease in perilesional edema and contrast enhancement [29].

Bevacizumab, an anti-vascular endothelial growth factor (VEGF) monoclonal antibody that inhibits the pro-inflammatory response, is increasingly used. Bevacizumab may elicit a dramatic response and significant MRI findings with rapid volume reduction on both contrast enhanced T1-weighted and T2-weighted MR images and reported mean reduction rates in follow-up of 62.017 and 48.58% respectively [30]. According to a recent meta-analysis, bevacizumab can be considered safe and efficacious for the treatment of radiation necrosis in brain metastases, however, the level of evidence was low [30].

In our study total edema volumes mirrored time course of MM load with a maximum reached after 2–4 months, a finding which is in accordance with former reports. Jardim et al. [31] investigated post-SRS edema in patients with MM and found a transient increase in mean edema volume at 3 months after RS that resolved by 6 months. It did neither correlate with adverse events nor the need for steroids. Another study investigated edema volumes after SRS in patients who concomitantly received ipilimumab and found edema quantities that mirrored MM volumes at 1.5, 3, and 6-month following SRS [9]. Interestingly, in patients concomitantly treated with ICI, there was no more measurable edema at 6 months follow-up and thereafter.

In a study that investigated perilesional edema, treatment with ipilimumab additionally to SRS was shown to improve tumor and edema volumes, whereas it was associated with a higher incidence of metastatic bleeds [32].

We found newly emerging metastatic bleeds in first follow-up 2 months after SRS that occurred in 37/169 irradiated MMs (= 21.9%) in 8 patients. MM hemorrhages with longer intervals (> 3 months) to SRS were limited to 4 MMs. Investigations that studied the rates of bleeding in MM post SRS reported of pretreatment hemorrhage rates of 21.8–23.7% and post treatment hemorrhage rates of 15.2–20.7% [33, 34]. Rate of pretreatment MM hemorrhage in our study was 5.9%. This low rate may result from early detection of MM in our patient collective, as patients had routine follow-up brain MRI scans after initial diagnosis of melanoma.

Other investigators studied bleeding rates in cerebral non-small cell lung cancer metastases combined treatment with ICI and found no increased rate of intratumoral hemorrhage in patients receiving concurrent ICI after SRS compared to patients with SRS alone [35] whereas in patients with MM, simultaneously administration of ipilimumab was reported with a higher incidence of lesion hemorrhage [32].

Differentiation between progression and therapy-induced changes such as pseudoprogression and radiation necrosis in cerebral metastases may be challenging on conventional MRI due to the lack of unequivocal distinguishing features. In addition, advances MR methods such as MR perfusion or MR spectroscopy have also limited diagnostic accuracy to differentiate between therapy-associated brain changes and real tumor progression [36–40].

For a retrospective analysis, it is easier to differentiate between real tumor progression and therapy-induced changes by analyzing the time course of the lesion evolution and regression. Hence, we followed-up the lesions after the first progression and only in case of further progression seen in the follow-up MRI scans; the finding was defined as real progression.

However, this course was not observed in the irradiated MM in our study. In contrast, the initial progression was defined as therapy-induced when the size decreased in the course of the disease, whereby there is no sharp MR imaging criterion dividing radionecrosis and pseudoprogression.

Beside conventional MRI and advanced MR methods, PET may be helpful in distinguishing real progression from pseudoprogression [41–45], but in our collective, PWI, MRS or PET-imaging was not performed. The MRIs were acquired to plan and to navigate the possibly immediately following Cyberknife therapy. Further prospective and large-scaled studies should use a multimodal design to evaluate, if the combination of MR morphological feature, hemodynamic and metabolic information have a real diagnostic gain to distinguish between progression and therapy-induced changes.

Our study is limited by its retrospective layout and small heterogenous patient population. Patients were treated in a CyberKnife SRS center with a large catchment area, thus patients had different preceding therapy regimens and presented at different stages of the disease at the time of SRS treatment. Furthermore, there were no available data from MM patients that were solely treated with SRS. A larger cohort could be helpful to uncover possible differences regarding PD-1 and CTLA-4 effects within this setting.

In conclusion, in early follow-up after SRS, findings suggestive of pseudoprogression were found in patients who concomitantly received ICI or ICI + TT. Our results may be explained by synergistic and potentiating cellular effects of ICI after SRS that have recently been shown in CNS neoplasms in experimental and clinical setup.

Even if the median survival time in patients with ICI was not reached in the follow-up period after SRS, there is a clear tendency for these patients for a longer survival, compared to the group of patients treated with TT.

## Data Availability

The datasets generated during the current study are available from the corresponding author on reasonable request.
